# Characterization and genome analysis of *Neobacillus mesonae* NS-6, a ureolysis-driven strain inducing calcium carbonate precipitation

**DOI:** 10.3389/fmicb.2023.1277709

**Published:** 2023-11-01

**Authors:** Rui Xu, Shuqi Zhang, Zhiwei Ma, Qingyan Rao, Yanling Ma

**Affiliations:** ^1^College of Life Science, Northwest University, Xi’an, Shaanxi, China; ^2^Shaanxi Provincial Key Laboratory of Biotechnology, Key Laboratory of Resources Biology and Biotechnology in Western China, Ministry of Education, College of Life Science, Northwest University, Xi’an, Shaanxi, China

**Keywords:** *Neobacillus mesonae*, urease activity, calcium carbonate, biomineralization, whole-genome sequencing, response surface methodology

## Abstract

In this study, a highly promising bacterium was isolated from sandstone oil in the Ordos Basin, named strain NS-6 which exhibited exceptional urease production ability and demonstrated superior efficiency in inducing the deposition of calcium carbonate (CaCO_3_). Through morphological and physiochemical characteristics analysis, as well as 16S rRNA sequencing, strain NS-6 was identified as *Neobacillus mesonae*. The activity of urease and the formation of CaCO_3_ increased over time, reaching a maximum of 7.9 mmol/L/min and 184 mg (4.60 mg/mL) respectively at 32 h of incubation. Scanning Electron Microscopy (SEM) revealed CaCO_3_ crystals ranging in size from 5 to 6 μm, and Energy Dispersive X-ray (EDX) analysis verified the presence of calcium, carbon, and oxygen within the crystals. X-ray Diffraction (XRD) analysis further confirmed the composition of these CaCO_3_ crystals as calcite and vaterite. Furthermore, the maximum deposition of CaCO_3_ by strain NS-6 was achieved using response surface methodology (RSM), amounting to 193.8 mg (4.845 mg/mL) when the concentration of calcium ions was 0.5 mmol/L supplemented with 0.9 mmol/L of urea at pH 8.0. Genome-wide analysis revealed that strain NS-6 possesses a chromosome of 5,736,360 base pairs, containing 5,442 predicted genes, including 3,966 predicted functional genes and 1,476 functionally unknown genes. Genes like *ure*A, *ure*B, and *ure*C related to urea catabolism were identified by gene annotation, indicating that strain NS-6 is a typical urease-producing bacterium and possesses a serial of genes involved in metabolic pathways that mediated the deposition of CaCO_3_ at genetic level.

## 1. Introduction

Microbially induced calcium carbonate precipitation (MICP) encompasses a range of processes that find significant applications in the fields of geotechnical and environmental engineering (Xu and Wang, 2023). Various studies have demonstrated that certain naturally occurring microbes are capable of rapidly producing thick mineral crystals with desirable gelling characteristics through their own metabolic activities, given appropriate living conditions, nutrition, and other external factors (Bhutange et al., 2020). Notably, the processes referred to MICP provide stringent control over the composition, structure, size and morphology of biominerals. So far, research on the application value of MICP has maintained a booming trend that includes the utilization of MICP for bio-concrete materials, reinforcement of rocks and soil, restoration of cultural artifacts, plugging of geological formations to enhance oil recovery and facilitate geologic CO_2_ sequestration, aimed with the advantages of environmental protection and ecological sustainability (Krajewska, 2018; Ortega et al., 2020; Al et al., 2022).

Various biological pathways have been proposed for MICP with extensive research conducted in recent years to understand the mechanisms and biochemical reactions involved (Zhang et al., 2023). Among these pathways, the most widely utilized method of MICP is based on the hydrolysis of urea by urease-producing bacteria. This approach offers several advantages, including rapid reaction rates, easy control of the process, high conversion efficiency, and more (Liu J. et al., 2021). Biologically induced mineralization occurs when urease-producing bacteria produce calcium carbonate (CaCO_3_), resulting in the gradual coating of bacterial cells as the number of CaCO_3_ crystals increases. This coating makes it difficult for the cells to transport and utilize nutrients required for metabolic activities, leading to their eventual death in solution (Kappaun et al., 2018; Naz et al., 2020). The type, size, and morphology of the mineralization products vary depending on the specific bacterial strains used under the same culture conditions. Therefore, the selection of appropriate ureolytic bacteria plays a crucial role in the success of solidification. In the past decade, various ureolytic bacterial species, including *Sporosarcina pasteurii*, *Citrobacter*, and *Enterobacter*, have been reported in MICP research (Kappaun et al., 2018; Keykha et al., 2019; Naz et al., 2020; Song et al., 2022). These literatures highlight how both bacterial species and abiotic factors can influence the mode and characteristics of CaCO_3_ formation, especially pH variations can lead to the precipitation of various CaCO_3_ forms. Alkaline conditions (pH > 8) promote the development of calcic carbonate, while acidic conditions (pH < 7) are more likely to yield calcite. Temperature also exerts influence over crystal shape and arrangement, wherein the deposition process is frequently expedited at higher temperatures, although lower temperatures can result in the formation of smaller crystals. High concentrations of calcium ions generally lead to increased CaCO_3_ deposition, and they can also give rise to crystals with distinct morphologies. Additionally, various strains of urease-producing bacteria may exert different effects on the morphology of CaCO_3_ crystals. Nutrient concentrations in the growth medium, like carbon and nitrogen and phosphorus sources, can impact bacterial growth and metabolism, consequently influencing the morphology of CaCO_3_ crystals. The effectiveness and availability of nucleation sites can further modify the morphology of CaCO_3_ crystals. The presence of the right nucleation site can induce growth in a specific direction, resulting in crystals with unique morphologies. Some bacterial strains may exhibit a tendency to produce large crystals, while others may yield small, uniformly shaped crystals. Most of the previous work to optimize MICP has focused on optimizing treatment conditions while considering the microbes as formulation ingredients, however, various properties of bacteria still need to be further explored accompanied by the molecular mechanisms underpinning MICP and the interplay between the abiotic factors. Thus, researchers should diligently search for new bacterial species to achieve efficient MICP, and the selection of an appropriate optimization scheme is crucial for enhancing large scale engineering practices of MICP technology.

The present study aimed to isolate and identify a strain named NS-6 that exhibited urease activity, and the morphological characteristics of CaCO_3_ and the influence of abiotic factors on CaCO_3_ formation by the isolated strain were investigated. The objectives of this research were as follows: (i) characterizing the NS-6 strain with high urease capability, (ii) conducting a preliminary assessment of CaCO_3_ formation, (iii) optimizing factors using the response surface method (RSM), and (iv) annotating the molecular mechanism through genome-wide sequencing and identification. These findings will provide valuable insights into the mechanisms and formation of carbonate minerals using a newly biological resource, thereby enabling wider industrial applications of MICP.

## 2. Materials and methods

### 2.1. Chemicals and culture media

A urea agar base plate was prepared for isolation and identification of urease-producing bacteria, consisting of 1.0 g/L peptone, 5.0 g/L NaCl, 2.0 g/L KH_2_PO_4_, 0.2% phenol red, 2.0 g/L urea, and 0.1 g/L glucose. Luria-Bertani (LB) broth was used for presentation and pre-culture, comprising 10.0 g/L tryptone, 5.0 g/L yeast extract, and 5.0 g/L NaCl. The mineral salt medium (MSM) contained (NH_4_)_2_SO_4_ at a concentration of 1.0 g/L, KH_2_PO_4_ at 7.0 g/L, K_2_HPO_4_ at 3.0 g/L, MgSO_4_•7 H_2_O at 3.0 g/L, and NaCl at 0.5 g/L (pH 7.0–7.2). For the calcium deposition tests, a mixture of 1,000 mL yeast powder (20.0 g/L), ammonium sulfate (10.0 g/L), and distilled water (pH 8.0) was used, following the NH4-YE culture medium advised by ATCC (Zhao et al., 2019). All culture media were adjusted to pH 7.0 and sterilized at 121°C for 20 min, except for urea and glucose that were filtered through a sterile 0.45 um membrane before being added to the urea agar base plate. All organic solvents and other reagents used in this study were of analytical grade.

### 2.2. Isolation and identification of urease producing bacterial strain

The ureolytic bacteria were isolated using the dilution plate and direct streak techniques. In detail, 10 mL of sandstone oil was added to 90 mL of MSM and cultured for 7 d at 30°C with 180 rpm shaking to enrich the bacteria. Then, 1 mL of the gradient-diluted enrichment solution from the MSM culture was coated onto a urea agar base plate and incubated for 24–48 h at 30°C. Colonies that exhibited the ability to hydrolyze urea and change the color of the agar base plate from orange to pink were selected for purification. A pure culture, named strain NS-6, was identified as having high levels of urease activity (Van et al., 2010; Gupta et al., 2022; Leeprasert et al., 2022). A negative control was prepared using *E. coli* DH5α, a known non-ureolytic bacterium. Strain NS-6 was then identified through morphological and biochemical characteristics, as well as 16S rRNA sequencing. Gram staining, nitrate reduction testing, and determination of other enzyme activities such as oxidase and catalase follow Bergey’s Manual of Determinative Bacteriology (Liu X. G. et al., 2021). The 16S rRNA gene sequence of strain NS-6 was amplified, the amplification of DNA fragments was carried out using the primer pairs including forward primer (CAGAGTTTGATCCTGGCT) and reverse primer (AGGAGGTGATCCAGCCGCA). The PCR amplification program consisted of an initial pre-denaturation step at 94°C for 5 min, followed by 35 cycles of denaturation at 94°C for 30 s, annealing at 55°C for 30 s, and extension at 72°C for 60 s, The final extension was performed at 72°C for 10 min. and compared to other sequences in GenBank through the Basic Local Alignment Search Tool (BLAST) to construct a phylogenetic tree using the neighbor-joining method in MEGA 7 software (Yoo et al., 2021; Uyar and Avcı, 2023). The NS-6 strain was stored at −80°C in 30% glycerol (Xu et al., 2022).

### 2.3. Urease activity and CaCO_3_ formation

Urease activity can be determined by measuring the change in conductivity per unit time. One unit of urease activity is defined as the amount of enzyme that hydrolyzes 1 μmol of urea per mL per minute, as determined by the method described by Yi et al. (2021) In brief, strain NS-6 was inoculated into NB-urea broth (0.3% nutrient broth and 2% urea, pH 8.0) at 30°C, and time-dependent urease activity was measured as follows. A mixture of 13.5 mL of 1.6 mmol/L urea solution and 1.5 mL of bacterial solution was prepared and incubated at 37°C for 5 min. During the cultivation process, the conductivity of the mixture (in mS/cm) was measured using a conductivity meter (DDS-307, Digital Conductivity Meter, China). Each treatment was repeated at least three times.

To investigate and quantify the formation of CaCO_3_ by strain NS-6, 3.0 mL of stationary phase bacterial cells (1% v/v) were inoculated into 300 mL liquid NH4-YE medium supplemented with 2% urea and 25 mM CaCl_2_. The mixture was incubated at 30°C and 200 rpm for 48 h. After incubation, 30 mL of the culture was sampled every 8 h. The samples were centrifuged at 5,000 rpm for 3 min to remove the supernatant, and the resulting precipitated CaCO_3_ was collected. The collected precipitates were then filtered and washed with absolute ethanol and deionized water to eliminate any residual cells and culture components. Subsequently, the precipitates were dried for 24 h at 80°C in a vacuum drying oven and weighed. To further analyze the CaCO_3_, it was washed with HCl solution, dried again, and weighed. The quantity of CaCO_3_ was determined by calculating the mass difference between the two dryings. The dried CaCO_3_ samples were preserved for future studies.

### 2.4. Micro-morphological characterization of CaCO_3_ crystals

A field emission scanning electron microscope (SEM, FEI Quanta 450, America) was employed to observe the micro-morphology of the mineralized products using an accelerating voltage of 1.5 kV. Concurrently, the sample surface was coated with a conductive gold film to facilitate electron flow and prevent bright spots in the captured images caused by electron aggregation, as the sample is non-conductive. Fourier-transform infrared spectroscopy (Nicolet iS50 FT-IR, Thermo Fisher, United States) was utilized to characterize the functional groups present in the mineralized products. The measured wavenumber range was from 4,000 cm^−1^ to 500 cm^−1^ at 25°C (Mohan et al., 2018). Additionally, X-ray diffraction analysis (XRD, Smartlab 9kw, Japan) was performed to determine the characteristic X-ray diffraction peaks of the mineralized products. The phase composition and crystal form of the X-ray diffraction peaks were analyzed using Jade 6 software. The scanning range was set at 5–75°, with a scanning step size of 0.02°. Thermogravimetry and differential scanning calorimetry (TG-DSC, TA Q600, America) were employed to investigate the simultaneous thermal analysis of the precipitates under a nitrogen atmosphere, with a heating rate of 20°C/min within the temperature range of 30°C to 950°C (Yi et al., 2021). All measurements were performed in triplicate.

### 2.5. Optimization of CaCO_3_ production by response surface method

The response surface method (RSM) was utilized to optimize the critical variables and their interactions in the formation of CaCO_3_ by strain NS-6. The independent variables, pH, urea concentration, and calcium ion concentration, were selected based on the results of the single-factor experiment (Supplementary Figure S1). Following the Box–Behnken central experimental design principle, a three-factor, three-level response surface analysis was conducted using a single-factor experimental design approach, with the optimal value as the central point and values above and below as the response surface experimental design levels (Wu et al., 2023). The experimental design, consisting of 17 treatments and 3 variables, was generated using Design-Expert V 10.0 software (Supplementary Tables S1, S2), with three replicates at the midpoint. By utilizing equation (1), a quadratic polynomial regression equation between the factor levels and response was derived.


(1) $$Y_{i}=b_{0}+\sum b_{i}X_{i}+\sum b_{ij}X_{i}X_{j}+b_{ii}X^{2}{}_{i}$$

In this study, *Y_i_* was used to represent the predicted response, while *X_i_* and *X_j_* were variables. The constant *b*_0_ was denoted as a constant term, *b_i_* referred to a linear coefficient, *b_ij_* indicated an interaction coefficient, and *b_ii_* represented a quadratic coefficient. Analysis of variance (ANOVA) was conducted to assess the statistical significance. The experiments were performed in 50 mL centrifuge tubes with transparent polypropylene material and a pointed bottom. The quantity of CaCO_3_ was determined using the gravimetric method. The calculation formula used was as follows:


(2) $$\mathrm{Quantity}\;{\mathrm{of CaCO}}_{3}=M_{1}–M_{2}$$

In this context, “*M*_1_” represents the amount of CaCO_3_ generated by strain NS-6, while “*M*_2_” stands for the quantity resulting from the abiotic treatments.

### 2.6. Complete genome sequencing of strain NS-6

High-quality genomic DNA from strain NS-6 was extracted using the Bacterial Genome Extraction kit (Tiangen, Beijing), following the manufacturer’s instructions. Subsequently, the obtained DNA was sent to Shanghai Meiji Bioinformatics Technology Co., Ltd. for whole-genome sequencing, with the aim of understanding the genetic-level molecular mechanisms underlying CaCO_3_ formation. Before being assembled into a contig using the hierarchical genome assembly technique (HGAP) version 2.2 of Canu, all clean reads underwent filtration and quality control based on the method described by Koren et al. (2017). CDS prediction, tRNA prediction, and rRNA prediction were performed using Glimmer, tRNA-scan-SE, and Barrnap, respectively, as described by Delcher et al. (2007). The predicted CDSs were annotated using sequence alignment techniques against the NR, Swiss-Prot, Pfam, GO, COG, and KEGG databases. Quick alignment of each set of query proteins with the databases allowed retrieval of gene annotations for the top matches (e-value <10^−5^). To analyze the genome of strain NS-6, the IslandViewer4 online website (Bertelli et al., 2022) was utilized, employing the IslandPick, IslandPath-DIMOB, and SIGI-HMM methods.

## 3. Results

### 3.1. Identification and characteristics of strain NS-6

**Figure 1:**
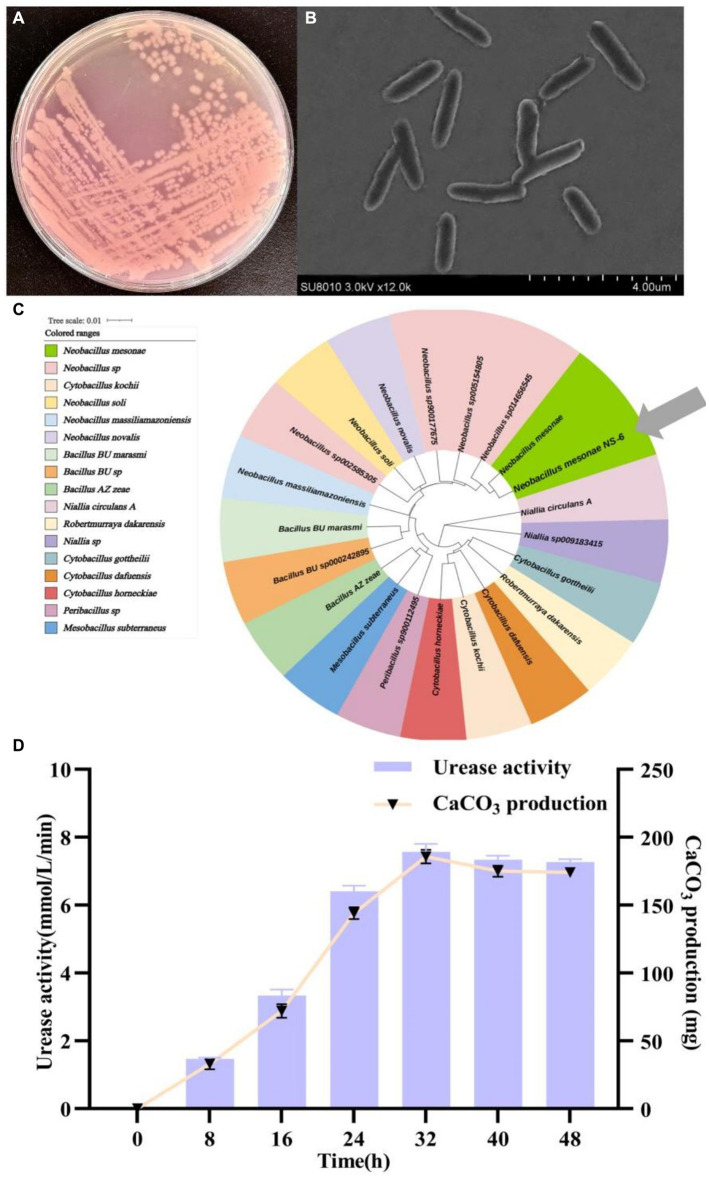
Morphology and identification of strain NS-6. **(A)** Typical colonies of strain NS-6 inoculated on a urea agar base plate, **(B)** Scanning electron microscope image of strain NS-6, **(C)** Phylogenetic tree constructed by adjacency method between strain NS-6 and 16S rRNA gene of related bacteria in MEGA 7.0 software, **(D)** Urease activity and calcium carbonate (CaCO_3_) production of strain NS-6 at 48 h of cultivation.

Numerous bacterial strains exhibiting morphological variations were observed to undergo a color change from orange to pink when inoculated on a urea agar base plate. Among these strains, a particular strain, designated NS-6, displayed a deep pink color due to its high urease activity. This strain was subsequently screened and identified. Following incubation on a urea agar base plate at 30°C for 48 h, individual colonies of the NS-6 strain exhibited a spherical shape with conical protrusions and ragged edges. Microscopic analysis revealed that the cell size was approximately 2.0 μm in diameter and rod-shaped (Figures 1A,B). The NS-6 strain exhibited positive activity in oxidase, catalase, nitrate reduction, and sugar fermentation tests shown in Table 1. Phylogenetic analysis further indicated that strain NS-6 shared a 99.8% homology with *Neobacillus mesonae* GCF-001636315.1 (Figure 1C). Additionally, Figure 1D demonstrated the changes in urease activity and CaCO_3_ formation over time for strain NS-6, which increased steadily and reached maximum values of 7.9 mmol/L and 184 mg (4.60 mg/mL) at 32 h of cultivation. Moreover, the deposition of CaCO_3_ was found to be quantitatively enhanced under conditions involving a calcium chloride concentration of 0.4–0.6 mmol/L, urea concentration of 0.8–1.0 mmol/L, temperature of 30°C, and pH range of 7–9, as depicted in Supplementary Figure S1. Those findings aligned with the optimal temperature and pH values reported in the earlier research works for the other urease-producing bacteria, and strain NS-6 exhibited significantly high urease activity at 32 h of cultivation compared to previously reported urease-producing bacteria (Qian et al., 2021; Song et al., 2022; Diez-Marulanda and Brandao, 2023).

**Table 1:** Physiological and biochemical characteristics of strain NS-6.

Characteristics	Strain NS-6	Characteristics	Strain NS-6
Colony color	Milky white	Citrate experiment	−
Cellular morphology	Long-rod	Lipid hydrolysis	−
Temperature for growth	20–45°C	Oxidase	+
pH for growth	6–9	Hydrogen peroxidase	+
NaCl for growth	0–100 g/L	Protease	+
Motility	+	Contact enzyme	+
Indole production	−	β-Galactosidase	+
Hemolysis	−	Lecithinase	−
Denitrification	+	Glucose	+
Gram stain	+	Maltose	+
Nitrate reduction	+	Lactose	+
Starch	−	Mannose	−
Gelatin hydrolysis	−	V-P test	−

### 3.2. Formation and structural characterization of CaCO_3_ crystals

**Figure 2:**
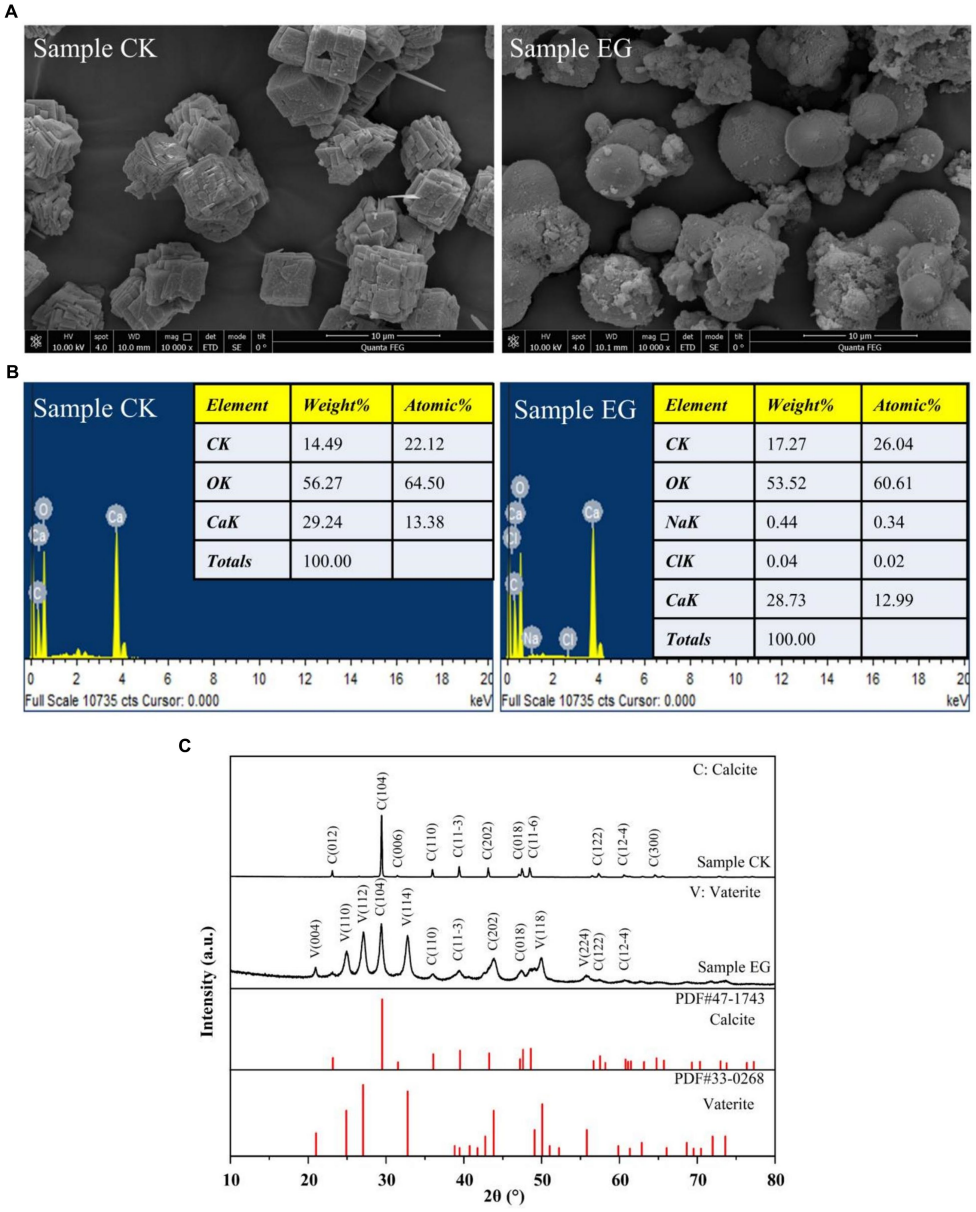
FESEM images **(A)**, EDS spectrum **(B)**, and XRD pattern of precipitated CaCO_3_ **(C)**. Sample CK was in the presence of *E. coli*, and sample EG was in the presence of strain NS-6.

**Figure 3:**
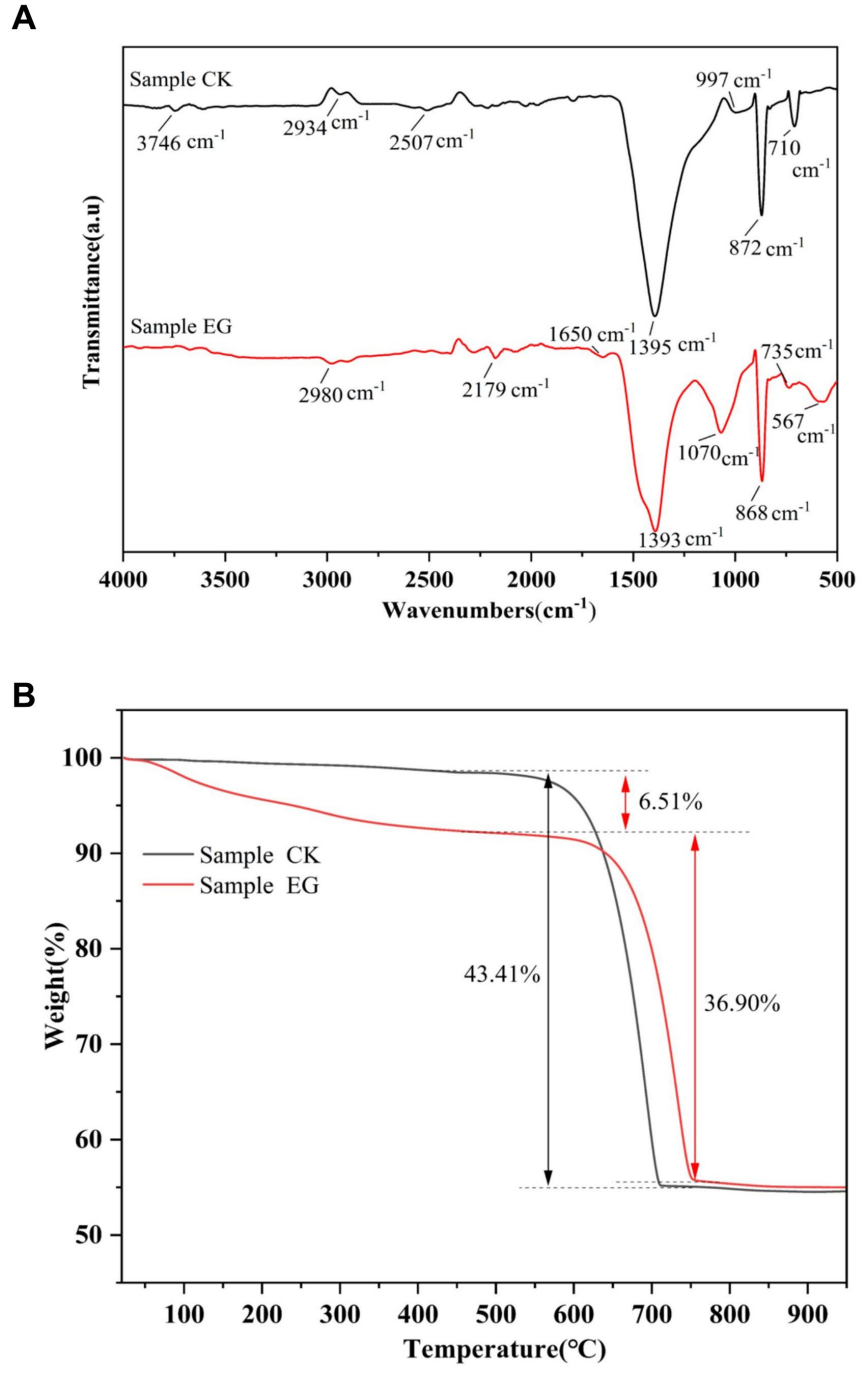
FT-IR spectra of precipitated CaCO_3_ **(A)**, and TG-DSC curves of precipitated CaCO_3_ **(B)**. Sample CK represented in the presence of *E. coli*, and sample EG represented in the presence of strain NS-6.

The formation of precipitated CaCO_3_ by strain NS-6 was investigated using NH4-YE culture medium, along with a negative control. The results showed that the CaCO_3_ precipitation by NS-6 exhibited irregular spherical compact large particles with a dense filling (Figure 2A). EDS analysis revealed the presence of Na, Cl, and other elements in addition to C, Ca, and O in the CaCO_3_ precipitate, indicating the presence of CaCO_3_ crystals (Figure 2B). XRD spectra confirmed the results of FESEM imaging, showing that the CaCO_3_ precipitation with the addition of strain NS-6 mainly consisted of calcite and vaterite (Figure 2C). Simultaneously, an analysis of calcite and spherulite content using XRD was conducted, and calculated their respective yield proportions by measuring the peak areas and masses of the respective minerals in the samples and underwent rigorous statistical analysis to ensure precision. The results revealed that calcite accounted for 31.7% in the sample, while spherulite constituted of 68.3%. Furthermore, the FT-IR spectra of the precipitated CaCO_3_ showed characteristic diffraction peaks at 710 cm^−1^ and 872 cm^−1^, which corresponded to the characteristic diffraction peaks of calcite. The characteristic diffraction peaks at 1,070 cm^−1^ and 868 cm^−1^ mainly corresponded to the spherulite characteristic diffraction peaks. Notably, the characteristic diffraction peak at 1650 cm^−1^ indicated the presence of a symmetric stretching of the carboxyl group (-COO-) in the protein secreted by strain NS-6 compared to the pure water system forming CaCO_3_ (Figure 3A). Thermogravimetric analysis revealed that the thermogravimetric curve of CaCO_3_ formed in the pure water system had a weight loss phase, with a total weight loss of 43.41% in the temperature range of 500–780°C, mainly due to the decomposition of CaCO_3_. On the other hand, the CaCO_3_ precipitated by strain NS-6 showed two weight loss stages on the TG curve (Figure 3B). The first weight loss phase occurred between 50 and 500°C, with a weight loss of 6.51%, attributed to the combustion and decomposition of bacterially secreted proteins adsorbed on the precipitated CaCO_3_. The second weight loss phase of 36.90% occurred in the range of 500–780°C, which was attributed to the decomposition of CaCO_3_.

### 3.3. Optimization of conditions induced CaCO_3_ precipitation

In this study, a Box–Behnken design based on response surface methodology (RSM) was employed to identify the key factors and their interactions that influence the induction of CaCO_3_ precipitation by strain NS-6. The results revealed that the pH (A), urea concentration (B), and calcium ion concentration (C) were the critical factors responsible for inducing CaCO_3_ precipitation, with a range of 130 mg to 196 mg. The corresponding precipitation rate ranged from 3.25 mg/mL to 4.90 mg/mL (shown in Supplementary Table S2). To model the quantity of CaCO_3_ precipitation induced by strain NS-6, a quadratic binomial regression equation (3) was fitted.


(3) $$\begin{array}{l} \mathrm{Yiled}\;{\mathrm{of CaCO}}_{3}\;\left(\mathrm{mg}\right)=+193.8+6.25\times A–1.5\times B\\ +16.5\times C–3\times A\times B+10.5\times A\times C–0.5\\ \times B\times C–32.15\times A^{2}–6.65\times B^{2}–11.15\times C^{2} \end{array}$$

**Figure 4:**
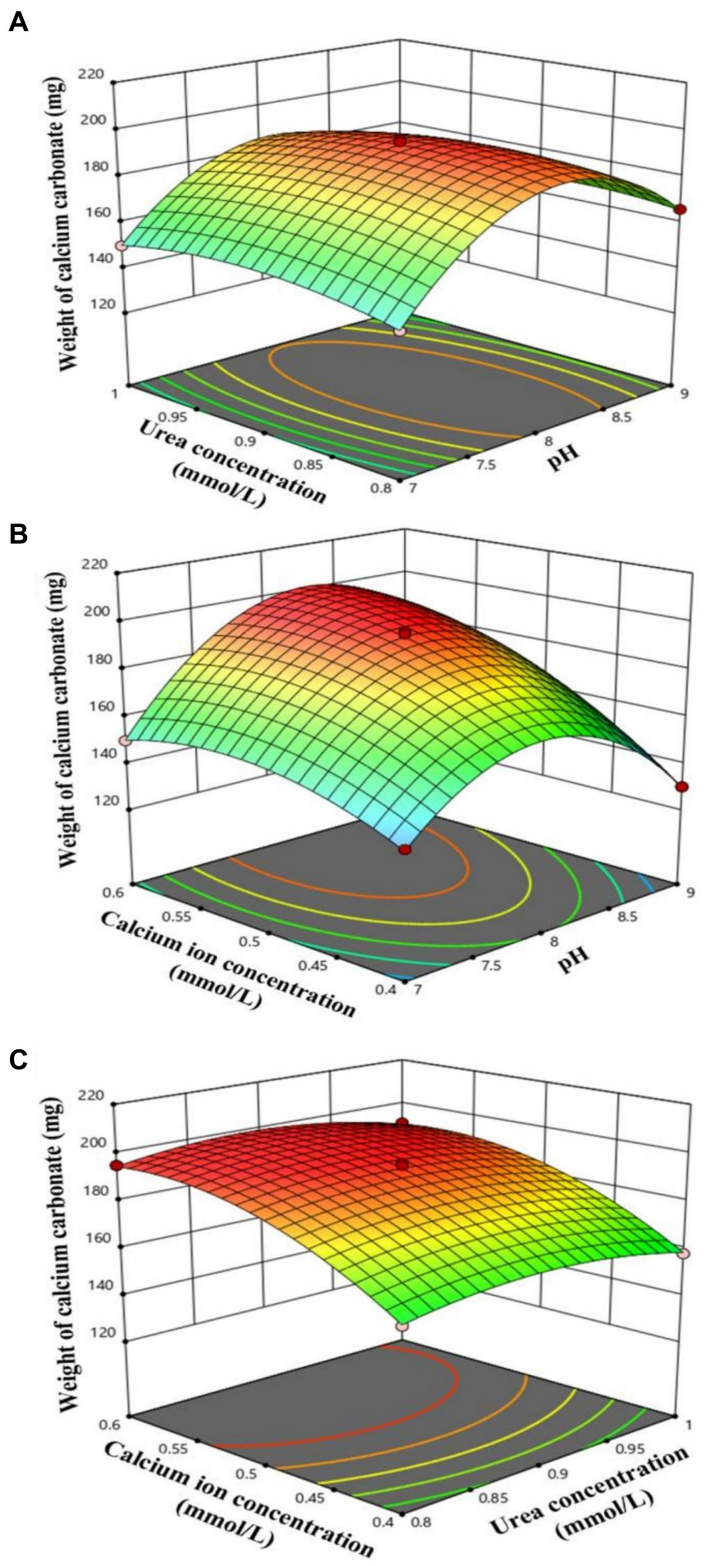
Response surface plots of the interaction effects of pH **(A)**, urea concentration **(B)** and calcium ion concentration **(C)** on the induction of CaCO_3_ precipitation by strain NS-6. **(A)** Interaction between pH and urea concentration, **(B)** Interaction between pH and calcium ion concentration, **(C)** Interaction between urea concentration and calcium ion concentration.

The ANOVA analysis of the quadratic polynomial model was presented in Table 2. The results show that the fitted terms were not statistically significant (*p* = 0.8192 > 0.05). However, the model had a high *R*^2^ value of 0.9984 and *R*^2^_Adj_ = 0.9964, indicating that the quadratic model accurately represents the relationship between the response and variables. Moreover, the results suggest that factors like pH (A) and calcium ion concentration (C), as well as the interaction term A × C and squared terms A^2^, B^2^, and C^2^, had significant effects on the formation rate of precipitation (*p* < 0.05) (Karimifard and Alavi, 2019). To further enhance our understanding of the results, a three-dimensional response surface was generated (Figure 4), which illustrates the impact of pH (A), urea concentration (B), and calcium ion concentration (C) on the quantity of CaCO_3_ precipitation while keeping other independent variables constant. From the 3D plot, it can be concluded that the variation in the surface due to pH (A) × calcium ion concentration (C) is greater than that due to pH (A) × urea concentration (B) and urea concentration (B) × calcium ion concentration (C) within the selected range of factors. The model predicts that a maximum amount of 193.8 mg (4.845 mg/mL) of CaCO_3_ precipitation can be achieved at pH 8.0, urea concentration of 0.9 mmol/L, and calcium ion concentration of 0.5 mmol/L. The reliability of the polynomial model equation is assessed through the *R*^2^ value, and its statistical significance is evaluated using the *F*-value. The *value of p*s of the model coefficients test the significance of the linear and squared effects of the influencing factors and their interaction effects.

**Table 2:** Strain NS-6 induced calcium carbonate (CaCO3) precipitation fitting quadratic polynomial model analysis of variance (ANOVA).

Source	Sum of squares	*df*	Mean square	*F*-value	*p*-value	
Model	8382.23	9	931.36	490.19	<0.0001	Significant
A-pH	312.5	1	312.5	164.47	<0.0001	
B-Urea concentration	18	1	18	9.47	0.0179	
C-Calcium ion concentration	2,178	1	2,178	1146.32	<0.0001	
AB	36	1	36	18.95	0.0033	
AC	441	1	441	232.11	<0.0001	
BC	1	1	1	0.5263	0.4917	
A^2^	4352.09	1	4352.09	2290.58	<0.0001	
B^2^	186.2	1	186.2	98	<0.0001	
C^2^	523.46	1	523.46	275.51	<0.0001	
Residual	13.3	7	1.9			
Lack of Fit	2.5	3	0.8333	0.3086	0.8192	Not significant
Pure Error	10.8	4	2.7			
Cor Total	8395.53	16				

### 3.4. Genome-wide sequencing and gene annotation

**Figure 5:**
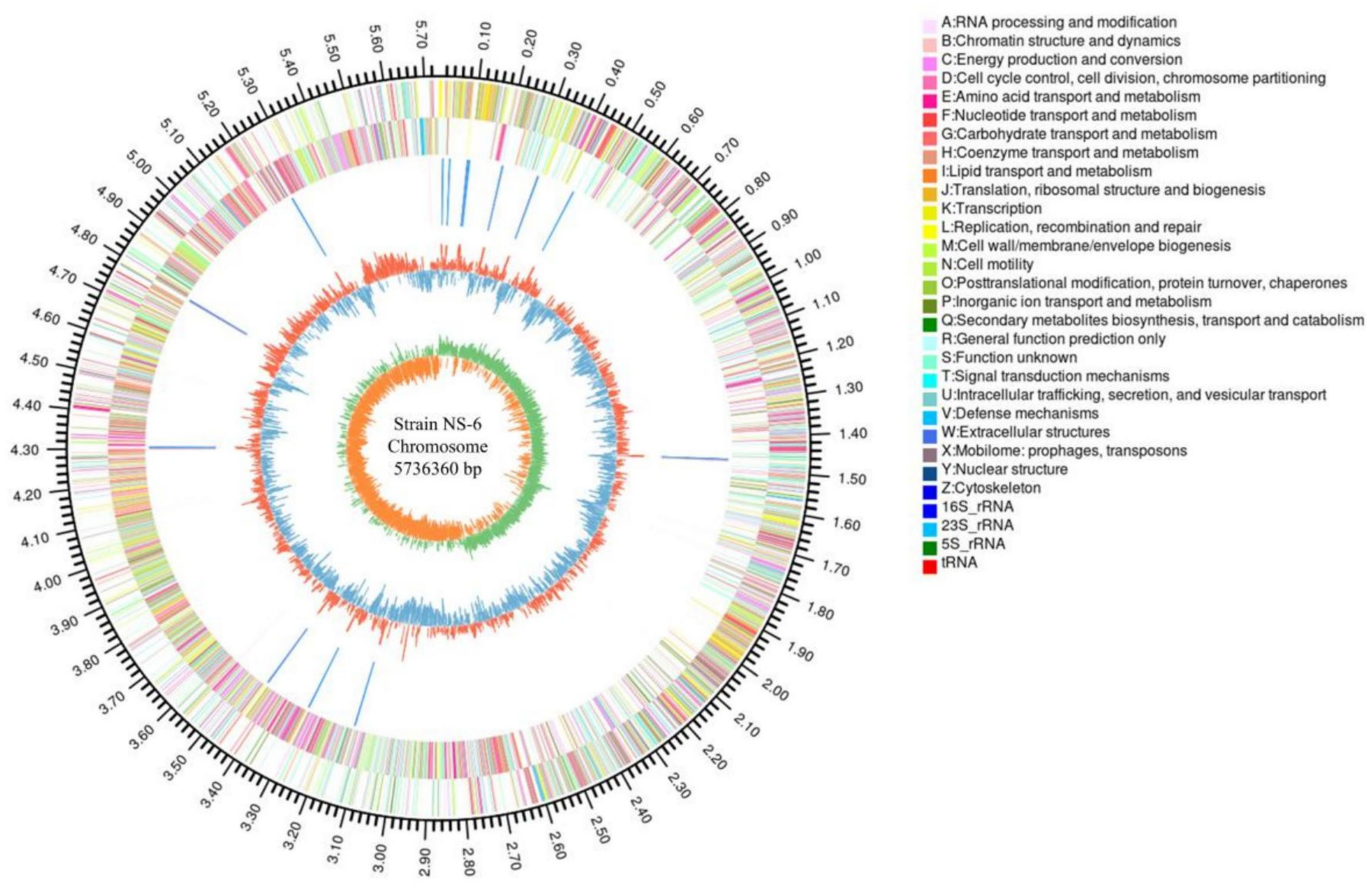
Graphical map of circular chromosome of the NS-6 strain.

**Figure 6:**
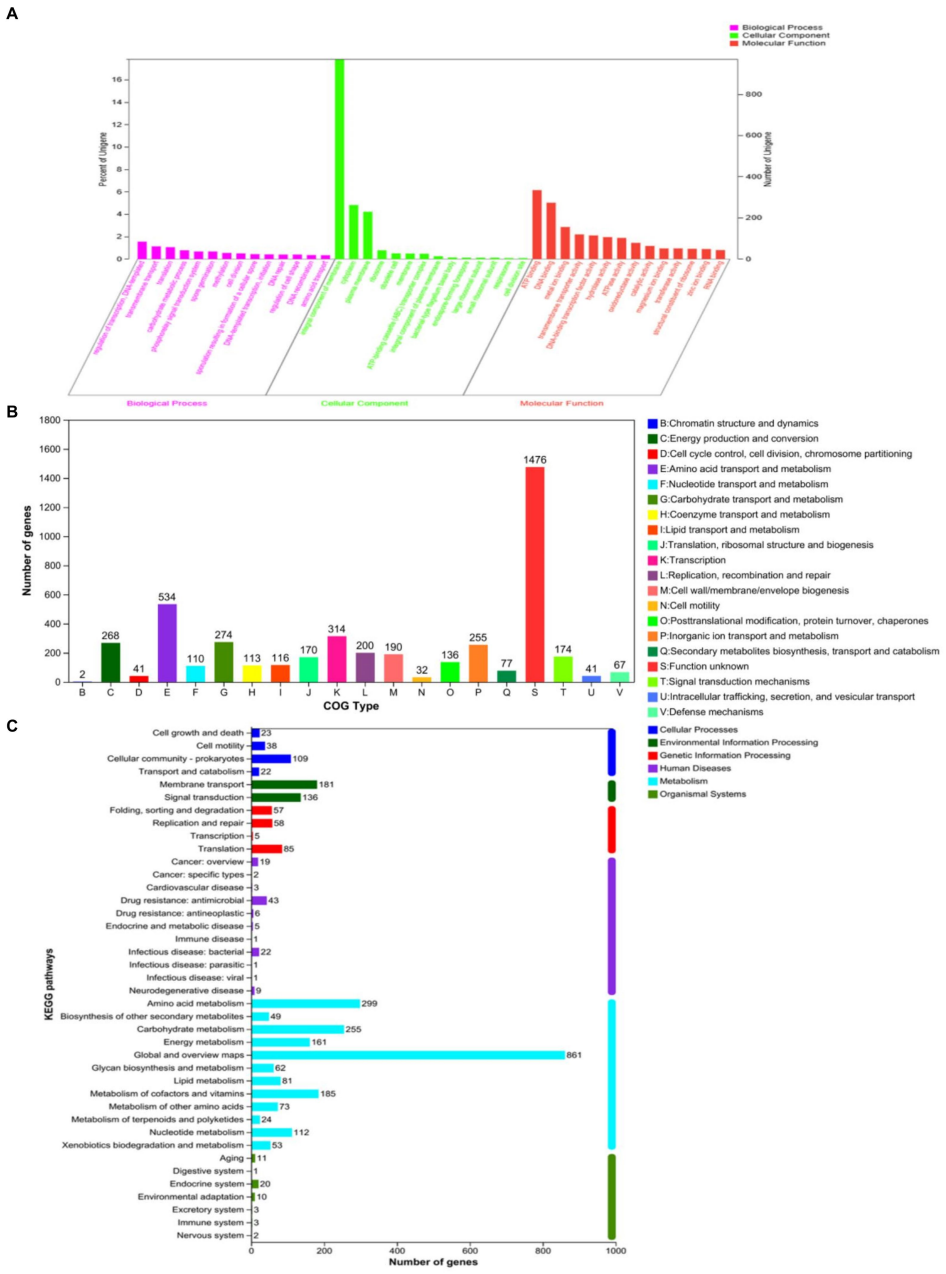
Number of genes associated with general functional categories, including **(A)** GO annotation map, **(B)** COG annotation map, and **(C)** KEGG annotation map of strain NS-6.

Aiming to better understand the genetic characteristics of strain NS-6, a genome-wide analysis was performed to reveal functional genes involved in the mineralization process. The genome of strain NS-6 consisted of a single chromosome spanning 5,736,360 base pairs (bp) with an average GC content of 40.32 mol% (Figure 5). Additionally, the genome contained 41 rRNAs and 110 tRNAs, and 13 genomic islands (Gls) were identified in strain NS-6 (Table 3 and Supplementary Figure S2). In terms of coding genes, there were a total of 2,676, 4,518, and 2,914 genes annotated for KEGG, COG, and GO databases, respectively (Figure 6). Notably, the genome analysis of strain NS-6 revealed the presence of urease-producing genes, namely *ure*A, *ure*B, and *ure*C, which encoded the γ subunit, β subunit, and α subunit of urease respectively, as well as genes encoded the urease accessory proteins UreD, UreE, UreF, and UreG (Table 4). This indicates that strain NS-6 is a typical urease-producing bacterium with a trimeric structure composed of two identical monomers, representative of urease enzymes, which was in agreement with earlier research works (Kappaun et al., 2018; Moro et al., 2022). These data through comprehensive whole-genome sequencing and functional annotation pave the way for future rational design of synthetic precipitator strains optimized for specific applications. The genome sequence of strain NS-6 has been submitted to the GeneBank database under accession number CP128196.1.

**Table 3:** General characteristics of NS-6 genome.

Feature	Chromosome
Genome size (bp)	5,736,360
GC content (%)	40.32%
Total genes	5,442
rRNA genes	41
tRNA genes	110
Other ncRNA	119
Genes with predicted function	3,966
Genes with unknown function	1,476
Genomic Islands	13
CDSs assigned to COGs	4,518
GenBank accession no.	CP128196.1

**Table 4:** Partial genes involved in urea catabolism.

Locus tag	KO ID	Gene Name	Size (bp)	Gene Description	Predicted Function
Gene3392	K01430	*ureA*	303 bp	Urease subunit gamma [EC:3.5.1.5]	Small subunit of (*ureABC*)_3_
Gene4915	K01430	*ureA*	303 bp	Urease subunit gamma [EC:3.5.1.5]	Small subunit of (*ureABC*)_3_
Gene3391	K01429	*ureB*	330 bp	Urease subunit beta [EC:3.5.1.5]	Small subunit of (*ureABC*)_3_
Gene4914	K01429	*ureB*	324 bp	Urease subunit beta [EC:3.5.1.5]	Small subunit of (*ureABC*)_3_
Gene3390	K01428	*ureC*	1713 bp	Urease subunit alpha [EC:3.5.1.5]	Large catalytic subunit of (*ureABC*)_3_
Gene4915	K01428	*ureC*	1713 bp	Urease subunit alpha [EC:3.5.1.5]	Large catalytic subunit of (*ureABC*)_3_
Gene3386	K03190	*ureD*	825 bp	Urease accessory protein	Urease accessory protein for Ni
Gene4909	K03190	*ureD*	825 bp	Urease accessory protein	Urease accessory protein for Ni
Gene3389	K03187	*ureE*	456 bp	Urease accessory protein	Urease enzyme active site formation
Gene4912	K03187	*ureE*	456 bp	Urease accessory protein	Urease enzyme active site formation
Gene3388	K03188	*ureF*	699 bp	Urease accessory protein	Urease accessory protein for Ni
Gene4911	K03188	*ureF*	687 bp	Urease accessory protein	Urease accessory protein for Ni
Gene3387	K03189	*ureG*	612 bp	Urease accessory protein	Urease accessory protein u*reG* parfois + *ureH*
Gene4910	K03189	*ureG*	612 bp	Urease accessory protein	Urease accessory protein u*reG* parfois + *ureH*
Gene1990	K01999	*livK*	1,188 bp	ABC transporter substrate-binding protein	ABC high affinity urea uptake system substrate-binding *livK*
Gene2721	K21405	*acoR*	2,514 bp	transporter substrate-binding protein	High affinity urea uptake system substrate-binding *acoR*
Gene3183	K01998	*livM*	1,026 bp	Branched-chain amino acid ABC transporter permease	Branched-chain amino acid transport ATP-binding protein *livM*
Gene3184	K01997	*livH*	867 bp	Branched-chain amino acid ABC transporter permease	ABC high affinity urea uptake system permease *LivH*
Gene4888	K01996	*livF*	717 bp	ABC transporter ATP-binding protein	Branched-chain amino acid transport ATP-binding protein *livF*
Gene5433	K01995	*livG*	780 bp	ABC transporter ATP-binding protein	ABC high affinity urea uptake system ATPase *livG*

## 4. Discussion

Urea hydrolysis, facilitated by urease-producing bacteria (UPB), is crucial for MICP. Among UPB, *Bacillus pasteurelli* and *Pseudomonas aeruginosa* have garnered significant attention from researchers across various disciplines (Qian et al., 2018). However, it is worth noting that different strains of bacteria can induce varied mineralization products even when subjected to the same culture conditions (Jiang et al., 2020; Ali et al., 2023). As a ureolytic agent, the process involves the use of urease-producing bacteria, and the most commonly utilized bacteria in this regard are arguably *Sporosarcina pasteurii*, and others (Kappaun et al., 2018; Pakbaz et al., 2018; Keykha et al., 2019; Naz et al., 2020; Yu et al., 2021; Song et al., 2022). Herein, *Neobacillus mesonae* strain NS-6 that possessed urease-producing capabilities and exhibited tolerance to alkaline environments, might potentially serve as a novel bacterial species for facilitating MICP. The spherical shape of CaCO_3_ crystals corresponded to vaterite, and the rhombohedral shape corresponded to calcite by strain NS-6, different from calcite mainly formed by other reported bacteria (Qian et al., 2021). Additionally, the expanded perlite particles exhibited numerous cavities, as observed through FESEM and XRD analysis. These cavities could potentially provide sufficient free oxygen for bacteria within the concrete, as well as offer attachment spaces for fixed bacteria to carry out their metabolic activities. Those results were coincident with some earlier research works, confirming that the specific morphology of formed crystals is influenced by differences in bacterial genera (Zheng et al., 2019, 2020; Wang Z. J. et al., 2022).

Even more to the point, the MICP process could be studied systematically by examining the optimal conditions for the growth and metabolism of common MICP-assisting bacteria (Liu et al., 2023). The parameters that have an effect on the course of CaCO_3_ precipitation and its efficiency, other than the type and concentration of bacteria/urease, primarily include the concentration of urea and calcium ions, as well as environmental factors such as temperature and pH value (Tang et al., 2020). RSM is a widely used optimization tool in the scientific community for studying the parameters that influence the process of MICP. By employing an appropriate experimental design, the number of required experiments can be reduced, allowing for the prediction of optimal performance conditions (Kumar and Kumar, 2021; Wang et al., 2021; Wang R. S. et al., 2022). To establish the relationship between the parameters and the quantity of CaCO_3_ precipitation, the variation of factor levels was tested using RSM. The squared and interaction terms were found to have significant effects on the response values. The *F*-values in the current study indicate that, within the selected test range, the ranking of the three factors in terms of their influence on the quantity of CaCO_3_ precipitation was calcium ion concentration > pH > urea concentration. This suggests that the optimal calcium ion concentration for CaCO_3_ formation is particularly crucial for the practical application of strain NS-6. These findings were consistent with previous studies that have shown the significant influence of calcium salt type on microbially-induced CaCO_3_ formation (Peng et al., 2022; Lv et al., 2023). It followed from the above that potential correlations between the variations in different factors were found during the experiments of the single influencing factor of MICP. Thus, not only the properties of the bacteria themselves, but also the environment and nutrients they were provided with had to be carefully considered when engineering bacteria for MICP and designing technological applications.

In addition to the optimal conditions for the formation of CaCO_3_ precipitation, the presence of genes involved in urea catabolism was identified in the whole genome of strain NS-6. All these results revealed that strain NS-6 contained all the genes involved in urea metabolism, and the fermentation *in vitro* also showed that this isolate had ureolytic activity, which were similar to the other urease-producing bacteria (Jin et al., 2017). Particularly, higher habitat pH correlated with higher copy numbers of *ure*C in environmental bacteria, according to previous research (Keykha et al., 2019). The same trend occurs with this isolate studied here, where duplication occurs in the isolate exposed to alkaline condition. With the acquisition of sequencing information, the isolated newly ureolytic strain NS-6 is preferred model to explore the relative importance of the metabolic pathways, regulatory mechanisms for urease production and its potential applications in industry and agriculture.

In conclusion, the present study has clearly shown that strain-specific precipitation of calcium carbonates from a newly isolate NS-6 occur during its optimum deposition condition and genome sequencing, and, based on the type of polymorph precipitated, this technology can be applied for various purposes.

## Data availability statement

The original contributions presented in the study are included in the article/Supplementary materials, further inquiries can be directed to the corresponding author.

## Author contributions

RX: Data curation, Methodology, Resources, Software, Validation, Visualization, Writing – original draft. SZ: Formal analysis, Investigation, Methodology, Software, Data curation, Writing – original draft. ZM: Formal analysis, Methodology, Software, Visualization, Writing – original draft. QR: Data curation, Formal analysis, Investigation, Methodology, Software, Validation, Writing – original draft. YM: Conceptualization, Funding acquisition, Methodology, Project administration, Supervision, Validation, Writing – review & editing.
