# Supplementary material for: Characterization and genome analysis of Neobacillus mesonae NS-6, a ureolysis-driven strain inducing calcium carbonate precipitation
Source: Front Microbiol. 2023 Nov 1;14:1277709. doi: 10.3389/fmicb.2023.1277709 (PMC10646308; doi:10.3389/fmicb.2023.1277709)
Supplement: Supplementary file 1 [file Data_Sheet_1.PDF]

Supplementary Materials

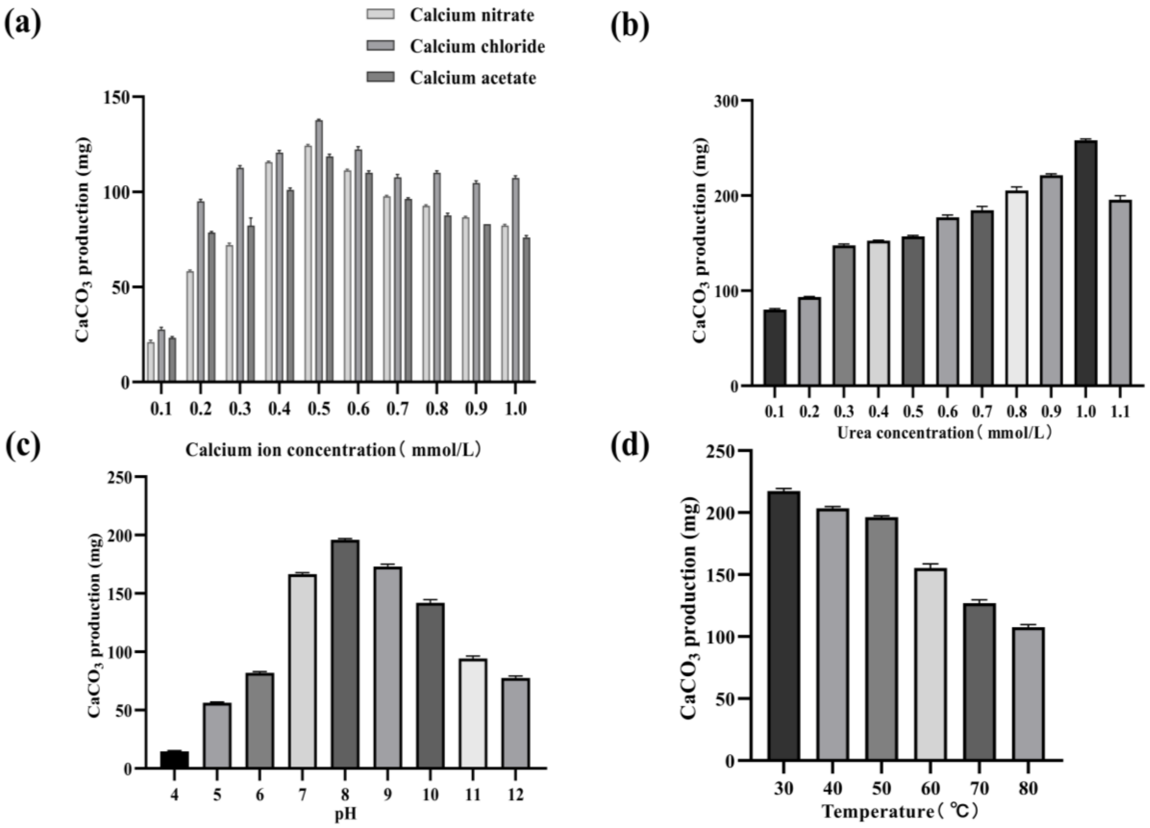

SUPPLEMENTARY FIGURE S1

Effects of calcium ion type and concentration **(a)**, urea concentration **(b)**, pH **(c)** and temperature **(d)** on the amount of calcium carbonate precipitation induced by strain NS-6.

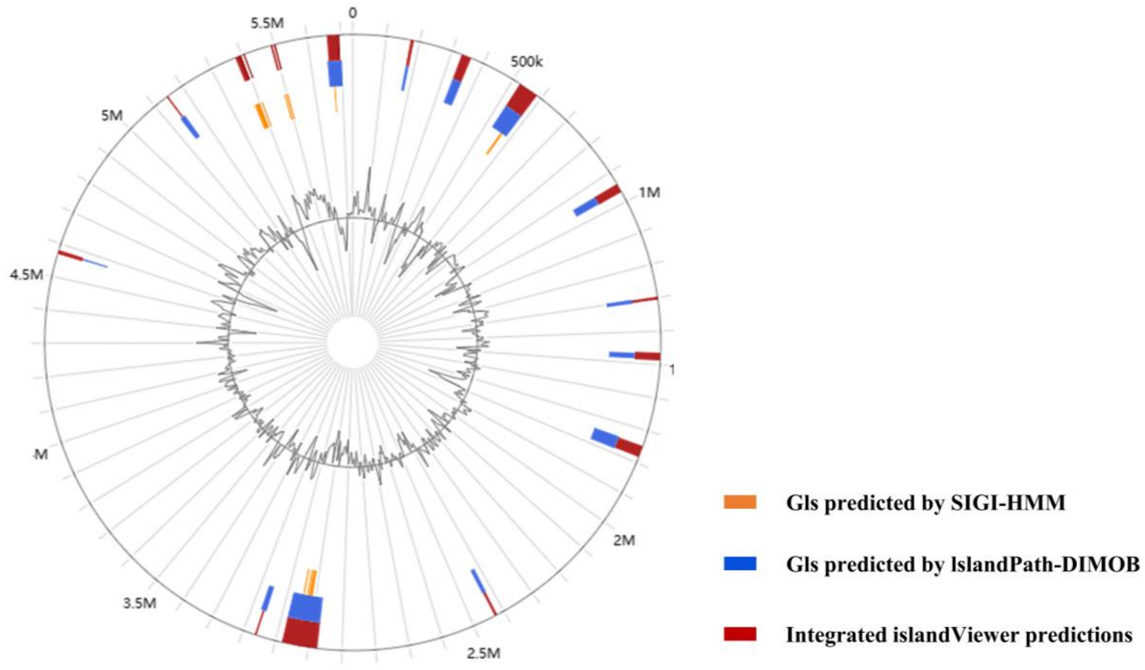

SUPPLEMENTARY FIGURE S2

Gene island group map of strain NS-6.

SUPPLEMENTARY TABLE 1 The code and levels of three independent variables used in Box-Behnken design.

Independent Variables	code	code levels of variables		
		-1	0	1
pH	A	7	8	9
Urea concentration(mmol/L)	B	0.8	0.9	1
Calcium ion concentration(mmol/L)	C	0.4	0.5	0.6

SUPPLEMENTARY TABLE 2 Box-Behnken random design matrix and its dependent variable response to strain NS-6 induced calcium carbonate precipitation.

Code	Factor A: pH	Factor B: Urea concentration(mmol/L)	Factor C: Calcium ion concentration(mmol/L)	$CaCO_3$ production
1	0	-1	1	195
2	0	0	0	196
3	0	1	1	191
4	0	0	0	195
5	-1	0	1	150
6	-1	0	-1	139
7	0	-1	-1	160
8	0	0	0	193
9	-1	1	0	150
10	1	0	-1	130
11	1	0	1	183
12	0	0	0	192
13	1	-1	0	166
14	0	1	-1	158
15	-1	-1	0	147
16	0	0	0	193
17	1	1	0	157
